# Muscle activity and head kinematics in unconstrained movements in subjects with chronic neck pain; cervical motor dysfunction or low exertion motor output?

**DOI:** 10.1186/1471-2474-14-314

**Published:** 2013-11-04

**Authors:** Harald Vikne, Eva Sigrid Bakke, Knut Liestøl, Stian R Engen, Nina Vøllestad

**Affiliations:** 1 Department of Health Sciences, Institute of Health and Society, University of Oslo, P.O. Box 1089, Blindern, NO-0317 Oslo, Norway; 2 Department of Informatics, University of Oslo, P.O. Box 1080, Blindern, NO-0316 Oslo, Norway

**Keywords:** Whiplash associated disorder, Persistent neck pain, Movement kinematics, Electromyography, Neck muscles, Movement smoothness

## Abstract

**Background:**

Chronic neck pain after whiplash associated disorders (WAD) may lead to reduced displacement and peak velocity of neck movements. Dynamic neck movements in people with chronic WAD are also reported to display altered movement patterns such as increased irregularity, which is suggested to signify impaired motor control. As movement irregularity is strongly related to the velocity and displacement of movement, we wanted to examine whether the increased irregularity in chronic WAD could be accounted for by these factors.

**Methods:**

Head movements were completed in four directions in the sagittal plane at three speeds; slow (S), preferred (P) and maximum (M) in 15 men and women with chronic WAD and 15 healthy, sex and age-matched control participants. Head kinematics and measures of movement smoothness and symmetry were calculated from position data. Surface electromyography (EMG) was recorded bilaterally from the sternocleidomastoid and splenius muscles and the root mean square (rms) EMG amplitude for the accelerative and decelerative phases of movement were analyzed.

**Results:**

The groups differed significantly with regard to movement velocity, acceleration, displacement, smoothness and rmsEMG amplitude in agonist and antagonist muscles for a series of comparisons across the test conditions (range 17 – 121%, all p-values < 0.05). The group differences in peak movement velocity and acceleration persisted after controlling for movement displacement. Controlling for differences between the groups in displacement and velocity abolished the difference in measures of movement smoothness and rmsEMG amplitude.

**Conclusions:**

Simple, unconstrained head movements in participants with chronic WAD are accomplished with reduced velocity and displacement, but with normal muscle activation levels and movement patterns for a given velocity and displacement. We suggest that while reductions in movement velocity and displacement are robust changes and may be of clinical importance in chronic WAD, movement smoothness of unconstrained head movements is not.

## Background

People having long-term musculoskeletal neck pain after motor vehicle accidents (Whiplash associated disorders - WAD) may have pronounced disability that affects daily living [1]. One of the most prominent clinical manifestations in persons with long-term WAD is cervical motor dysfunction [2], signified by altered neck muscle activation and reduced peak motor output. For example, measures of peak performance such as low load isometric endurance and maximum voluntary isometric contraction force of neck- and shoulder girdle muscles are known to be reduced in chronic WAD [3-9]. For relatively unconstrained dynamic head movements, kinematic performance variables such as peak velocity are also lower than in healthy participants [10,11] and the peak head movement displacement is typically reduced in people with chronic WAD [10-17]. Collectively, these alterations reduce the functional capacity of the head and neck in people with chronic WAD.

The causes for the reductions in peak kinematic performance variables in chronic WAD are less clearly understood. It has been shown that the activation of neck muscles is altered in chronic WAD as compared with healthy subjects [5,18] and such changes may potentially affect the head kinematics. However, these results were obtained from studies of isometric neck muscle contractions at low to moderate muscle forces [5,18]. It is therefore possible that these observations are not directly applicable to dynamic head movements. In a study of movement kinematics in chronic WAD and control participants, Sjölander [19] found no difference in peak movement velocity when taking movement displacement into consideration. Because of the close relationship between the kinematic parameters of displacement and peak velocity [20], it is therefore possible that reductions in head displacement may contribute to the reductions in peak head movement velocity in chronic WAD.

In addition to measures of peak performance, the regularity or smoothness of neck movements in people with chronic neck pain are reported to be reduced [11,15,19,21]. Smooth movements are characterized by approximately bell-shaped and unimodal velocity profiles [22], while movements of reduced smoothness exhibit multi-peaked, irregular velocity profiles containing a series of accelerative and decelerative phases. Such irregular movement patterns have been suggested to be a consequence of motor control disturbance in people with persistent WAD [11,19]. In a previous study, the irregularity of movement was shown to be strongly related to both the movement velocity and displacement across a series of different head movements in healthy participants [23]. Since it is known that people with chronic WAD perform with both lower movement velocity and less displacement compared with controls, it raises the question of whether the reduced smoothness of movements observed in people with chronic WAD may simply be caused by altered movement velocity and displacement and not altered movement control strategies.

In this study we compared head kinematics and muscle activation in relatively unconstrained neck movements at three different speeds in participants with and without chronic WAD. In addition comparisons were made taking both movement velocity and displacement into consideration.

## Methods

### Participants

We examined 15 patients (six men and nine women) suffering from chronic WAD (> 6 months), classified as grade 2 according to the Quebec Task Force classification [24] and which started less than 72 hours after the motor vehicle accident. In addition, six men and nine women matched with the WAD group for sex and age (± 5 years) served as controls. The following exclusion criteria were used: WAD grade 3–4, pregnancy, age ≤ 18 or ≥ 60 years, unsettled insurance claims, systemic inflammatory diseases, neurological disorders, tremor, regular usage of analgesics and strongly reduced vision/blindness or auditory defects. All patients were recruited from a local rehabilitation clinic and examined by a specialist in physical medicine or neurology and a manual therapist before inclusion. Descriptive data for the participant groups are given in Table 1. The study was approved by the Regional Committee for Medical and Health Research Ethics, and all participants signed an informed consent form for participation in the study in accordance with the Helsinki declaration.

**Table 1:** Descriptive data for the chronic WAD and control groups (six men and nine women in each group)

**Variable**	**WAD**	**Control**
Age (yrs)	40.1 (8.7)	38.7 (8.8)
Height (cm)	170.5 (8.5)	173.2 (7.6)
Weight (kg)	78.3 (13)	75.8 (13.9)
BMI (kg/m^2^)	26.9 (4.2)	25.1 (3.2)
Head mass (kg)	4.33 (0.34)	4.53 (0.30)
Hand grip strength, dominant (kg)	45.3 (11.4)	50.1 (12.6)
non-dominant (kg)	41.8 (11.8)	47.3 (12.1)
SF-36, PCS (0–100)	33.4 (9.7)**	54.4 (5.0) (n = 14)
MCS (0–100)	45.3 (15.0)*	55.2 (4.8) (n = 14)
Duration of symptoms (months)	22 (98)	-
Pain intensity, pre-test (1-10)	3.1 (1.4)	-
post-test (1-10)	5.6 (2.0)#	-
NDI (0–50)	21.7 (5.6)	-
FABQ, W (0–42)	22.3 (10.2)	-
PA (0–24)	10.5 (4.5)	-

Results are average (SD). For the duration of symptoms the results are median (interquartile range). Statistically significant difference from the control group; * p < 0.05, ** p < 0.0005. Statistically significant difference from the pre-value within group; # p < 0.0005.

Abbreviations; *BMI* body mass index, *SF-36* short form-36, *PCS* physical component summary, *MCS* mental component summary, *NDI* neck disability index, *FABQ* fear avoidance beliefs questionnaire, *W* work, *PA* physical activity.

### Overview and procedures

In this study we examined the movement performance of unconstrained head movements in the sagittal plane at three different speeds in participants with and without chronic WAD. Head movement performance was assessed with respect to displacement, velocity and acceleration and measures of movement smoothness and symmetry. Neck muscle activity was measured by means of surface electromyography (EMG) of agonist and antagonist muscles. Descriptive measures of anthropometry and overall strength were taken as they may affect the outcome variables in the study. All experiments were performed in a standardized laboratory setting. All participants completed one separate training session in order to familiarize themselves with the testing procedures 1–2 weeks in advance of the experiment. Tests were completed in the following order: 1) maximum handgrip strength, 2) evaluation of pain intensity, 3) tests of head movements and 4) re-evaluation of pain intensity. Participants were given pauses ad libitum.

### Descriptive data

#### Anthropometrics and grip strength

The participants’ body height (cm) and weight (kg) were measured and the body mass index (kg/m^2^) calculated. Head volume was measured for men and women as described by McConville [25] and Young [26], respectively and a density of 1.05 kg/l [27] was used to estimate the head mass. As a measure of overall muscle strength [28,29], hand grip strength was tested on a hand dynamometer (Model 78010, Lafayette Instruments) adjusted individually. The base rested on the first metacarpal and the bar on the second to fifth medial phalanx. Participants were told to squeeze as hard as possible and to maintain the force for three to four seconds. Each hand was tested two to three times (60 s inter-test pause) and the highest value was used in further analysis.

#### Self-reported questionnaires

A numerical rating scale (1–10) was used to assess subjective pain intensity in the neck and head region at the time of measurement, where 1 represents absence of pain and 10 the worst imaginable pain. Participants rated the pain intensity in the head and neck immediately before and after completion of the neck movement testing. The neck disability index (NDI) was used as a measure of physical disability due to neck pain [30]. Fear of movement and of movement-related pain in work and physical activity in general was measured using the fear avoidance beliefs questionnaires (FABQ) work and physical activity subscales [31]. In line with previous studies in people with neck pain [32], we modified the FABQ by replacing the word back with neck. The health-related quality of life was assessed by the generic Short-Form Health Survey 36 (SF-36), version 1 [33]. The questionnaire’s mental and physical component summary measures were calculated using Norwegian normative values [34], where the population norm score is defined as 50 ± 10 (SD) for both scales.

### Head movements

#### Movement directions

**Figure 1:**
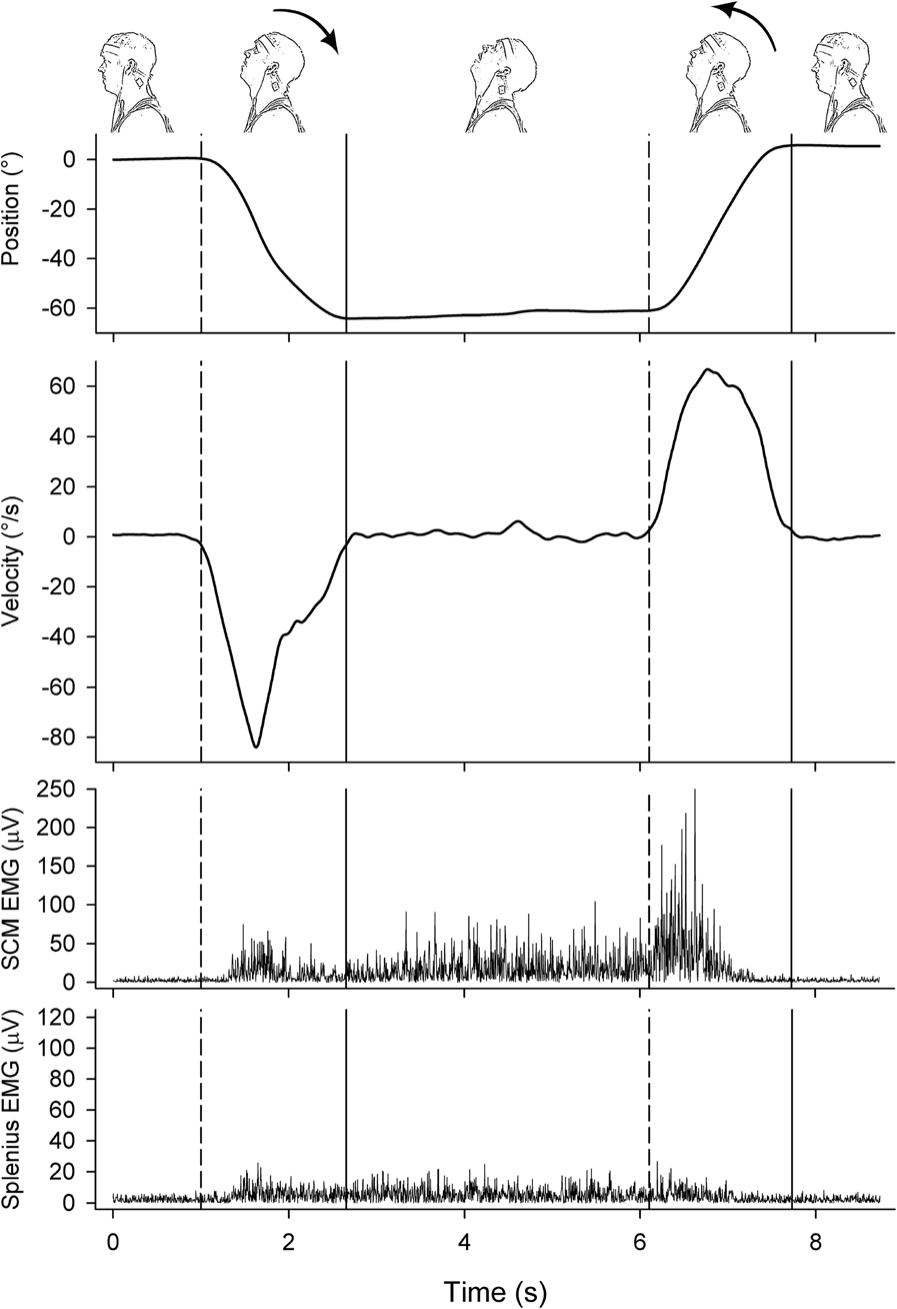
**Example of two movement directions (extension from neutral position (EFN) and flexion back to neutral position (FBN), top row) completed at the preferred speed condition and the accompanying data for position (second row), angular velocity (third row) and rectified electromyography (μV) for the left sternocleidomastoid (SCM) (forth row) and splenius muscles (bottom row).** Note the differences in Y axis scaling between muscles. Dashed and solid vertical lines depict start and stop of movements, respectively. The control participant scored about average for the kinematics and electromyographic amplitude for both movements.

Four head movements were completed in a custom designed chair as previously reported [23]. While sitting the participants were instructed to position themselves in their individual resting position with respect to their head when looking straight forward at a wall that was approximately 120 cm in front of them. This was defined as their neutral head position (NP). When sitting in this position, a 15 mm diameter dark blue dot was applied at the participant’s individual focus point on the wall as an individual reference for the NP. The participants completed four head movements in the sagittal plane, each corresponding to approximately half of their full range of motion: forward flexion from NP (FFN), extension back to NP (EBN), extension from NP (EFN) and flexion back to NP (FBN). Participants were asked to move their head and neck as far as possible when starting from the NP and to stop at NP when starting from the fully flexed or extended position. The order of the direction of movements was randomized for each participant. Examples of two movement directions are shown in the top of Figure 1.

#### Movement speeds

The participants were tested in three different speed conditions. First, the participants were instructed to complete all movement directions in a pace corresponding to what they perceived as their normal speed, which was termed preferred speed (P). Thereafter they were instructed to move at about half of their preferred speed, termed slow speed (S) and finally with their maximum speed (M). To put as little constraint on the movements as possible, the participants were not given any feedback on their performance during testing. The participants were allowed to practice the movement directions and speed conditions before the test started and usually 2–4 trials were performed. The participants completed 3 trials per speed condition for each direction and these were averaged for further analysis. All trials were accepted, except if the participants expressed that the movements deviated from what they had intended to do, then retrials were performed.

### Kinematics

#### Data sampling and analysis

Position data were sampled using an electromagnetic motion tracker (Liberty, Polhemus Inc.) at 240 Hz as previously described in detail [23]. The signals, analyzed off-line in MatLab, were filtered using a quintic Woltring spline with a cutoff frequency of 6 Hz. The quintic spline additionally defines the higher order derivatives (velocity, acceleration and jerk). Movement onset and offset were defined to be 4% of the peak angular velocity [23]. If the signal fluctuated across this 4% threshold, the final crossing was used for the offset. Movement duration and displacement were defined as the time and angular position difference between the movement onset and offset, respectively. The overall smoothness of the movement was calculated as the normalized jerk cost (NJC) according to Teulings [35]. To further examine the regularity of the movement, the number of submovements was counted as described by Ketcham [36]. Movement symmetry for movements consisting of one submovement was measured by the velocity profile symmetry index [37], taken as the time to peak velocity divided by total movement time. Values less than or above 0.5 indicate asymmetry in the velocity profile. For movements consisting of more than one submovement, the spatial occurrence of the submovements was calculated as the relative number of submovements started in each of the two movement halves. The reliability of this setup and kinematic outcome measures have been previously examined and shown to be acceptable [23].

### Electromyography

#### Muscles and sensor placement

Electromyographic signals were sampled bilaterally from the sternocleidomastoid (SCM) and the splenius muscles. The signals were detected and pre-amplified 10x using single differential active surface sensors consisting of two parallel 10 x 1 mm silver electrode bars (DE-2.1, Delsys Inc.). The sensor placement on the SCM muscle were marked on the skin using published suggestions [38], then examined by ultrasound imaging using a 10 MHz, 5 cm linear array probe (Vingmed, General Electrics) and adjusted if necessary. After locations were established, the skin was first shaved and then firmly rubbed and washed with 70% isopropyl alcohol using electrode prep pads and the electrodes fastened using double adhesive tape. A 50 mm diameter ground electrode was placed over the left olecranon. From now on the SCM will be referred to as agonist during flexion movements and antagonist during extension movements. The opposite will be done for the splenius muscle. See Additional files 1 and 2 for a more detailed description.

#### Data sampling and analysis

The pre-amplified signals were passed to a main amplifier (Bagnoli-16, Delsys), amplified 1000x, band-pass filtered between 20–450 Hz with a built-in analog filter, AD-converted (NI-DAQ 6220, National Instruments) and sampled at 1 KHz. EMG signals were offset-adjusted and the running root mean squares (rms) were calculated in window lengths of 50 ms with 49 ms window overlap using an EMG software package (EMGworks 3.7). Baseline EMG was subtracted from the reference- and movement rmsEMG signals. EMG epochs covering the entire movement as defined by the kinematic start and stop procedures ± 200 ms were subsequently normalized to the median rmsEMG accomplished at reference contractions (see Additional files 1 and 2 for descriptions) and further analyzed. A few trials containing large spiked artifacts were excluded. The signal obtained during movement was separated into two epochs; one beginning at the start of movement as defined above for the position data and ending at the time point of peak velocity was defined as the accelerative phase, and one epoch starting at peak velocity and ending at the stop of movement was defined as the decelerative phase. The signals of bilateral muscle pairs were averaged for further analysis. Due to very low EMG activity during the S and P speed conditions for the gravity-assisted movements (EBN and FFN), the EMG was analyzed for the movements completed against gravity, i.e. the flexion and extension back to neutral position (FBN and EBN).

### Statistics

Graphical displays were used to assess the distribution of the data. After log-transformation of right-skewed distributions, the data were found to be approximately normally distributed. The WAD and the control group were compared using independent samples t-tests. Differences within the groups between speed test conditions within a given movement direction were examined using analysis of variance for repeated measures.

Possible effects of velocity and displacement on the NJC and number of submovements were also examined by comparing groups using general linear models with velocity and displacements as covariates. As both displacement and velocity affects the EMG amplitude [39], the rmsEMG data were compared using the same model and covariates. Since peak movement velocity is strongly related to the displacement of movement [20], we also compared groups for peak velocity and acceleration at the M speed conditions using displacement as a covariate.

To test for differences between groups in the spatial distribution of submovements and of the velocity profile symmetry index we used a mixed factor general linear model with participants as random factor. Bivariate correlations were performed using Pearson’s correlation coefficient. The scores of NDI, FABQ and pain intensity were analyzed against movement displacement, peak velocity and acceleration and rmsEMG amplitude of the P and M speed condition for all movement directions. Tests are two-sided and p-values less than 0.05 were considered statistically significant. Statistical analyses were performed using the SPSS 18 and JMP 9.0 statistical packages.

## Results

### Group characteristics

The chronic WAD group and the control group were not significantly different with respect to age, anthropometrics or grip strength (Table 1). The WAD group displayed statistically significantly lower values of both the physical and mental component summary scales of the SF-36 than the control group did (p-values < 0.05). According to the scale of Vernon [30], the mean absolute NDI score of 22 for the WAD group was in the upper part of the range (15–24) defining moderate physical disability due to neck pain. The fear avoidance levels of the WAD group related to physical activity and work were also moderate.

### Kinematics

**Figure 2:**
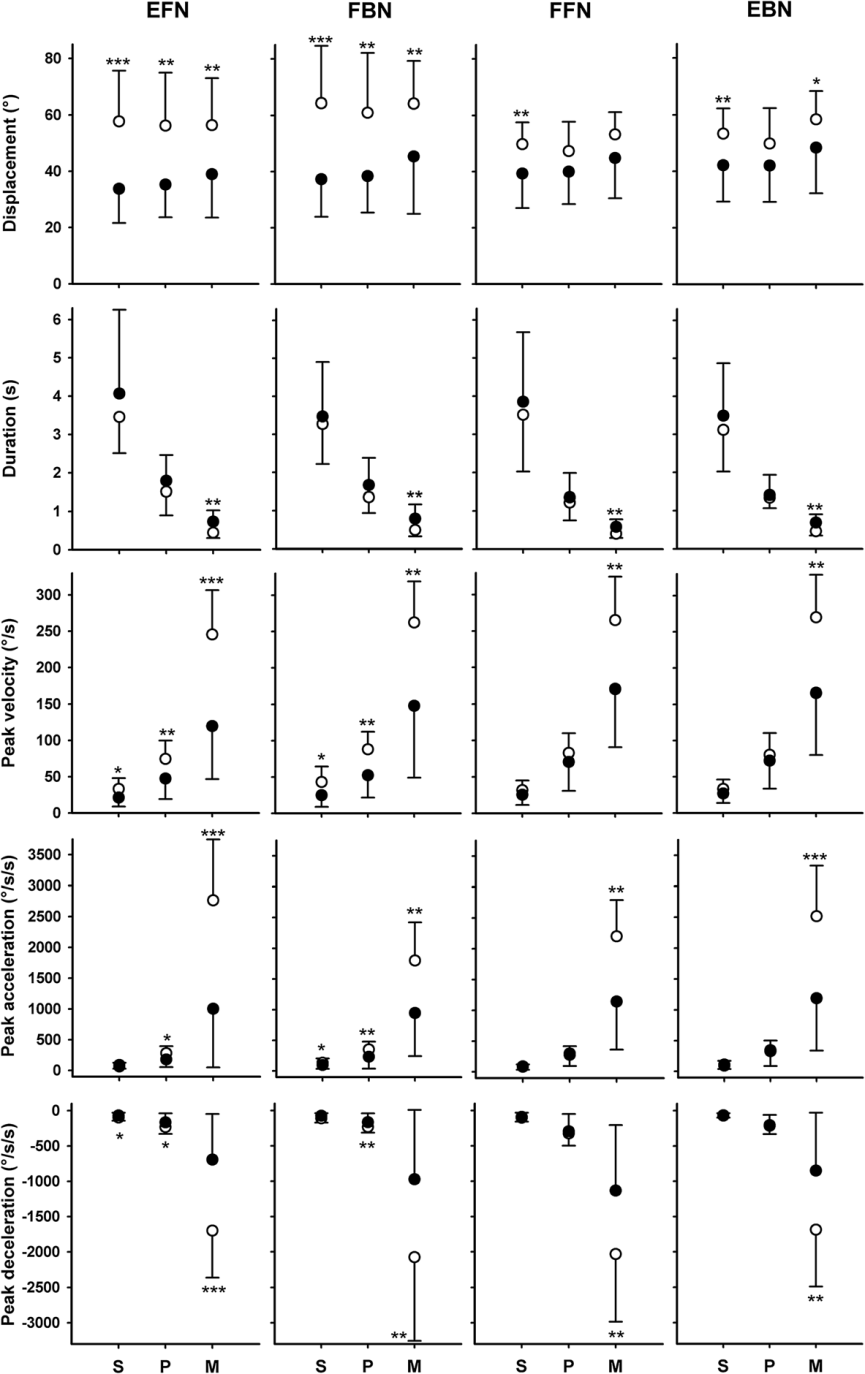
**Average (± SD) kinematic data for the three speed conditions (slow (S), preferred (P) and maximum (M)) in the four movement directions (columns EFN (extension from neutral position), FBN (flexion back to neutral position), FFN (flexion from neutral position) and EBN (extension back to neutral position)) for the WAD (filled circles, n = 15) and control (open circles, n = 15) groups.** Significant group differences; * p < 0.05, ** p < 0.01, ***, p < 0.0005. Y axis scaling is identical across the rows.

All movement variables for head and neck kinematics showed differences between participants with- and without chronic WAD and the detailed results are presented in Figure 2 and Table 2. In summary, the mean values for displacement were numerically lower for the WAD group compared to controls in all comparisons across all movement directions; in 8 of 12 cases the differences were statistically significant (p-values < 0.05). Similar results were also found for both peak and average velocity as 16 of 24 comparisons were significantly lower for the WAD group (p-values < 0.05). The peak acceleration and deceleration were significantly lower in the WAD group in 14 of 24 cases (p-values < 0.05). Peak and average velocity and peak acceleration and deceleration were significantly lower in the WAD group compared to the control group for all movement directions for the M speed condition (p-values < 0.01). Also, for the preferred test speed, the peak and average velocity and peak acceleration and deceleration were lower in the WAD group for the EFN and FBN movement.

**Table 2:** Average (SD) angular velocity (°/s), normalized jerk cost (a.u.) and number of submovements for the three speed conditions in the four movement directions for the chronic WAD (n = 15) and control groups (n = 15)

		**EFN**		**FBN**		**FFN**		**EBN**	
		**WAD**	**Control**	**WAD**	**Control**	**WAD**	**Control**	**WAD**	**Control**
Av. vel.	S	10.8 (6.7)**	18.2 (7.8)	13.4 (9.0)*	22.4 (10.6)	12.5 (6.7)	16.7 (7.7)	14.2 (6.9)	19.1 (7.2)
(°/s)	P	23.6 (12.2)**	40.0 (13.2)	27.6 (15.4)**	45.9 (12.7)	37.3 (21.1)	42.8 (13.9)	35.3 (18.9)	38.5 (11.1)
	M	66.6 (44.9)***	133.2 (42.6)	76.1 (56.3)**	136.8 (33.7)	87.1 (44.7)**	139.6 (36.7)	81.8 (45.6)**	131.6 (30.1)
NJC	S	1541 (2933)	335 (269)	824 (1502)	319 (250)	969 (1318)	467 (521)	704 (1171)	275 (287)
(a.u.)	P	134 (173)	58 (43)	95 (134)	42 (24)	79 (117)	45 (30)	74 (107)	47 (19)
	M	33 (22)	27 (14)	42 (40)**	19 (5)	24 (12)	20 (11)	30 (15)	25 (8)
Subm.	S	14.6 (12.4)	9.4 (3.9)	11.3 (7.0)	9.3 (5.7)	14.6 (12.6)	11.4 (7.5)	12.9 (8.8)	9.5 (6.8)
(no.)	P	3.9 (2.9)	2.4 (1.6)	3.2 (2.4)*	1.6 (0.8)	2.6 (2.3)	1.8 (1.0)	1.9 (1.3)	1.5 (0.6)
	M	1.5 (0.7)*	1.1 (0.2)	1.4 (0.9)	1.0 (0.0)	1.1 (0.2)	1.0 (0.1)	1.3 (0.5)	1.0 (0.1)

Statistically significant difference from the control group; * p < 0.05, ** p < 0.01.

Abbreviations; *NJC* normalized jerk cost, *a.u.* arbitrary units, *Subm*. submovement, *no*. number, *Av.vel.* average velocity, *NP* neutral head position, *EFN* extension from NP, *FBN* flexion back to NP, *FFN* flexion from NP, *EBN* extension back to NP, *S* slow movement speed, *P* preferred movement speed, *M* maximum movement speed.

The differences between groups in peak velocity and acceleration at the M speed conditions were also evident after using displacement as a covariate (p-values < 0.05).

**Figure 3:**
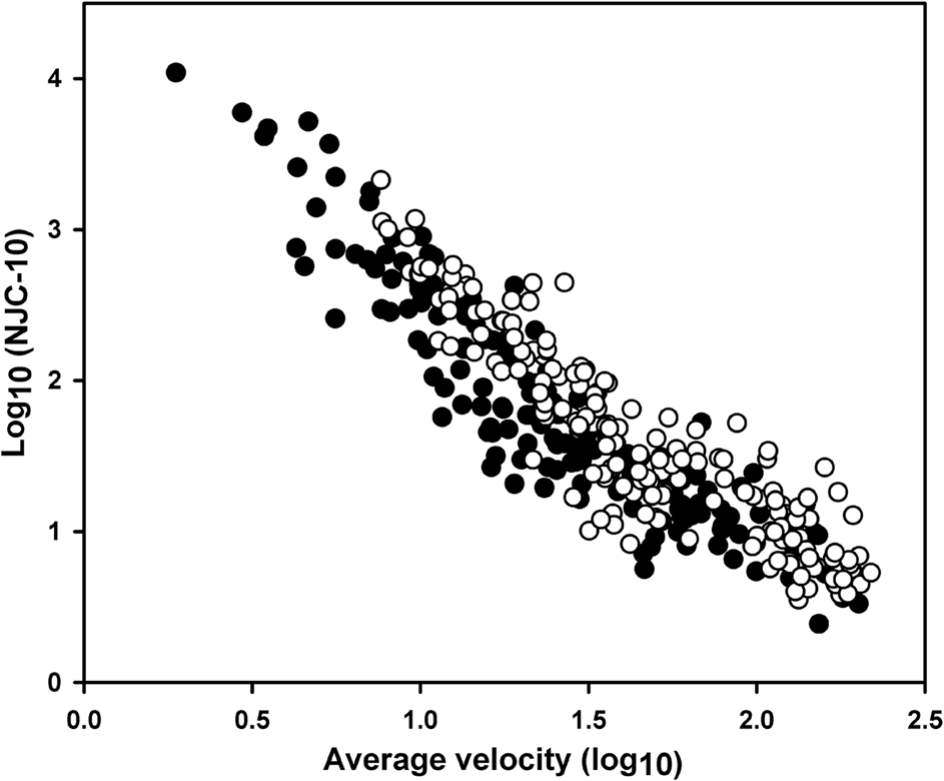
**Relationship between average velocity (log**_**10**_**) and normalized jerk cost (NJC-10) (log**_**10**_**) across all speed conditions for all movement directions pooled for the WAD (filled circles, n = 15, 180 trials) and the control (open circles, n = 15, 180 trials) groups.**

**Figure 4:**
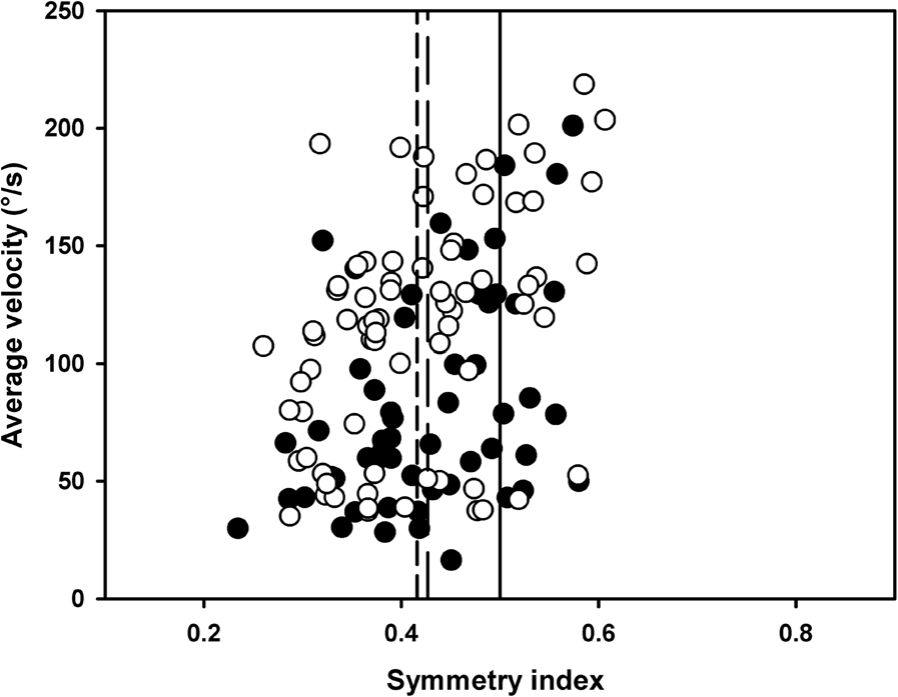
**Velocity profile symmetry index (scale) versus average angular velocity (°/s) for the WAD (filled circles, n = 15, 55 trials) and control (open circles, n = 15, 73 trials) group.** Solid vertical line indicates symmetric movements (value 0.5). Long and short dashed lines indicate the average values (0.427 ± 0.082 and 0.416 ± 0.087, p = 0.81 for group differences) for the WAD and control groups, respectively.

Mean values for NJC and number of submovements were numerically consistently higher in the WAD group than the control group (Table 2), although the large variation between individuals implied that the difference was significant (p < 0.05) only for one and two test conditions, respectively. However, when displacement and velocity were used as covariates, no differences were found between groups for either NJC or number of submovements at any test velocity for any movement direction (Figure 3). We found no statistically significant differences between groups in the spatial distribution of submovements in either of the two movement halves (both p = 0.91); for the WAD group, 54 ± 17% of the submovements started in the first half of the movement displacement compared to 57 ± 18% in the control group. Nor did we find any significant difference in the velocity profile symmetry for the movements consisting of one submovement only (p = 0.81; Figure 4). In summary, we detected no difference in either the smoothness or the symmetry of movement between the two groups.

### Electromyography

In the sitting position prior to testing, the absolute baseline rmsEMG amplitude (μV) was not significantly different between groups for any muscle studied (Table 3, p-values > 0.25). Similarly, we found no group differences in absolute rmsEMG amplitude (μV) values during the reference contractions for the muscles (p-values > 0.11).

**Table 3:** Average (SD) rms electromyographic amplitude (μV) for the sternocleidomastoid (SCM) and splenius muscle at rest and at reference contractions (rest values subtracted) for the chronic WAD (n = 15) and control (n = 15) participants

	**Rest**		**Reference contractions**	
**Muscle**	**WAD**	**Control**	**WAD**	**Control**
SCM	2.75 (0.26)	2.74 (0.19)	40.12 (17.36)	51.79 (21.61)
Splenius	2.73 (0.69)	2.52 (0.17)	11.42 (5.12)	10.95 (3.24)

There were no statistically significant differences between the groups.

**Figure 5:**
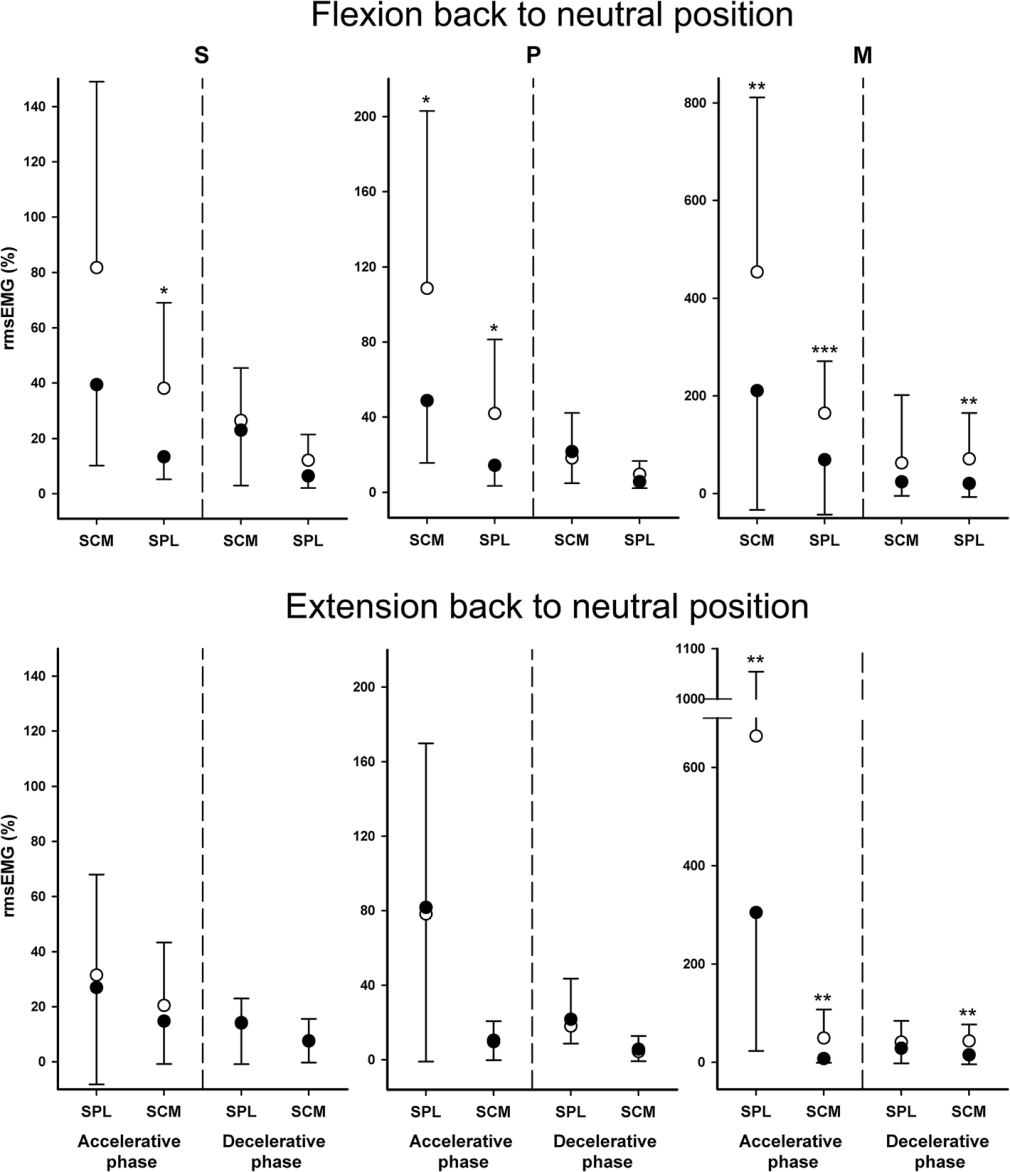
**Electromyograpic (rmsEMG) amplitude (%) of the sternocleidomastoid (SCM), splenius (SPL) muscle at the accelerative and decelerative phase of the three different speed conditions (S (slow), P (preferred) and M (maximum)) for the flexion back to neutral position (upper panels) and extension back to neutral position (lower panels) movements for the WAD (filled circles, n = 15) and control (open circles, n = 15) groups.** The data are average and error bars standard deviation. Statistically significant group differences; * p < 0.05, ** p < 0.01, ***, p < 0.0005. Note the differences in Y axis scaling among the separate test conditions.

There was an overall effect of increased movement velocity as assessed by the separate test speed conditions on both the agonistic and antagonistic muscle rmsEMG amplitude for the two phases of both movement directions analyzed (14 of 16 comparisons were statistically significant, p-values < 0.05). For the acceleratory phase of the M speed condition for all movement directions, the relative rmsEMG amplitude of both the agonistic and antagonistic muscles were significantly lower for the WAD group compared with the controls (p-values < 0.01, Figure 5). For the decelerative phases of these movements, only the antagonistic muscles displayed lower amplitude for the WAD group (p-values < 0.01). For the FBN direction, reduced muscle activity in the WAD group was also found for the accelerative phase at the P speed condition in both the agonist and antagonistic muscles and at the S speed condition for the antagonistic muscles (p-values < 0.05). No group differences were found for the EBN direction at either the S or P speed (p-values > 0.38).

When the EMG data was compared between groups while controlling for velocity and displacement, all statistically significant differences between groups in rmsEMG amplitude for the agonist and the antagonist muscles vanished (p-values > 0.18) with one exception: the activity in the antagonistic SCM muscle was significantly different between groups in the acceleratory phase of the EBN movement at the M speed condition (p < 0.05).

### Association between kinematics, EMG and self-reported data in the WAD group

We did not find any significant relationships between the self-reported pain intensity at baseline and the kinematics or rmsEMG amplitude (r value range -0.44 to 0.42, all p-values > 0.10). The NDI correlated only with the antagonistic splenius rmsEMG amplitude during the accelerative phase of the P speed at the FBN movement (r = -0.59, p < 0.05).

The FABQ physical activity subscale correlated significantly with displacement for the P and M speed condition at the EFN and FBN movement directions (r value range -0.55 to -0.65, p-values < 0.05), and with peak velocity and acceleration at the M speed condition for the EFN and FBN movements (r value range -0.52 to -0.59, p-values < 0.05).

For the FBN movement the FABQ physical activity subscale correlated with the rmsEMG amplitude for the agonist muscle at both the accelerative and decelerative phase for the P and M speed tests (r value range -0.67 to -0.77, p-values < 0.01).

### Supplementary data

Participant groups were additionally examined using extra loading (+25% of head mass) at the P speed test only. The results for each group were similar to that of the unloaded P speed condition and the data are therefore shown in the Additional file 1.

## Discussion and conclusions

The findings in the present study of generally reduced displacement, peak acceleration, deceleration and velocity at the maximum (M) speed conditions for the WAD group compared to controls are in close agreement with a number of previous studies examining participants with chronic WAD [10-17]. For the EFN and FBN movements we also found reduced displacement, peak acceleration, deceleration and velocity at the preferred (P) movement speed to be different between groups, which are consistent with previous observations [14]. Thus, even though the WAD participants held a large reserve capacity in movement velocity as displayed by the result of the M speed test, they preferred to move at a lower velocity than the controls for the P speed test. Differences in movement velocity and displacement at both the maximum and preferred speed between the participants with chronic WAD and controls therefore seem to be a general and robust finding. The pain level of the WAD group increased significantly following the experiment, which seems to be a common finding in studies involving physical exertion of various intensity by persons with musculoskeletal disorders [40,41].

The lower peak velocity and acceleration at the M speed condition for the WAD group compared with the controls also persisted after controlling for movement displacement. There are several possible explanations for such a group difference including muscle morphological and muscle activation strategies. Since we neither measured single cell- nor gross muscle area in the present study, we cannot exclude muscle atrophy as a possible factor for reductions in peak acceleration or velocity. However, when using magnetic resonance imaging, previous studies have not detected atrophy of the total cervical muscle cross-sectional area in chronic WAD participants area as assessed by case–control studies [42-44] or in a 6-month follow-up study of WAD participants [45]. Thus, group differences in neck muscle size seem to have limited explanatory strength for differences in head kinematics in the present study. As the maximum shortening velocity of a muscle is strongly dependent upon its fiber type composition [46], an increase in the proportion of slow muscle fibers of the neck muscles could possibly reduce the head movement velocity. However, the result from an uncontrolled, cross-sectional study of participants with neck pain of various etiologies on the contrary indicates a possible, minor increase in the fast fiber type direction [47]. Also, the reported type 1 fiber proportion in the neck muscles from the participants with post-traumatic etiology in the study of Uhlig [47] is almost identical to that found in presumably healthy participants [48]. Thus, it seems that the most reasonable explanation for the altered kinematics in the WAD participants would be the muscle activation patterns. We found a large reduction in agonist rmsEMG amplitude at the M speed test in the WAD group compared with the controls, which supports the interpretation that the reductions in peak acceleration and velocity at the M speed tests are a result of lowered muscle activation. This reduced activation found in the M speed test may in turn be partly explained by fear of pain since we found significant negative associations between the FABQ physical activity component and both peak acceleration, velocity and agonist muscle rmsEMG amplitude in the M speed test. Moderate relationships between fear-avoidance beliefs and displacement [49] or force [50] have also been reported previously in subjects with neck pain. It is therefore possible that peak exertion is voluntarily reduced to sub-maximal levels partly because of pain and/or fear of pain. It is also possible that the peak muscle activation is reduced because of motor inhibition by pain afferents [51,52]. The reduction in neural drive at the M speed test may also be a function of both sub-maximal voluntary activation and motor inhibition.

The electromyographic activity of the agonist and antagonist muscles during both the acceleratory and deceleratory phases of the movement was on the other hand not different between groups when movement velocity and displacement were taken into consideration. Thus, the results of the present study suggest that for a given velocity and displacement of dynamic neck movements, the chronic WAD participants activated the involved muscles to the same degree as healthy controls. Although we are not aware of any studies that have examined neck muscle activation during dynamic unconstrained neck movements in WAD participants, one study examining participants with chronic non-specific neck pain and controls reported no group difference in EMG amplitude of the cervical erector spinae muscles at a duration-controlled EBN movement [53]. These data are in keeping with our data, but they contrast somewhat with previous trials using isometric contractions [5,18]. For example, Schomacher [5] found the average EMG activity (μV) of the semispinalis muscle to be significantly lower in participants with chronic WAD compared with controls during circular isometric contractions at standard force levels (15 and 30 N). Their data strongly indicate that other neck muscles, either synergistic or antagonistic, must have altered their activity in parallel with that of semispinalis to generate the resultant forces. There were no indications of such a rearrangement of intermuscular activation patterns in the present study since we found no difference in the rmsEMG amplitude between groups after controlling for velocity and displacement for either the splenius or the SCM muscles in any movement direction. It is possible that such differences may be attributed to the muscle contraction types examined and/or the relative voluntary effort used in tests.

The smoothness and regularity of movement did not differ between groups after the movements were controlled for velocity and displacement. As indicated in Figure 3, the WAD group in fact tended to move more smoothly for a given velocity than the controls. This contrast between groups can however be explained by differences in movement displacement, since a movement of a given velocity becomes smoother by reductions in displacement [23] and this has not been taken into account in the figure. Thus, across a large range of head movement velocity, chronic neck pain due to WAD does not seem to alter the smoothness of movements compared with controls. This finding therefore contradicts the conclusions drawn from previous studies that did not control for movement velocity that implicitly suggested that movement smoothness in unconstrained movements is altered *per se* for participants with chronic neck pain [11,15,19,21]. Also, to further assess the dynamic movement strategies, we also examined the symmetry of movements and the spatial occurrence of submovements and found no significant group differences. These findings indicate that chronic WAD neither lead to a rearrangement of intermuscular activation patterns nor resultant movement patterns *per se* in relatively simple, unconstrained dynamic head movements. This conclusion is also somewhat in contrast to other studies that have found increased irregularity of velocity-controlled and constrained motion paths in chronic WAD compared with healthy participants [54,55]. The head movements used in both these studies were highly spatially constrained by the imposition of visual trajectory tracking [54,55]. As several [56-59], although not all [60] studies have found reduced eye-movement control in chronic WAD, it is possible that the different conclusions made may be related to the dependence on visual involvement in the movement tests used.

A key question relates to the external validity of the study. Are the two groups of participants comparable for variables not related to neck pain? And are the chronic WAD participants representative for patients with chronic WAD group I and II? While there were no group differences with respect to descriptive data for age, anthropometrics or grip strength, the WAD group scored significantly poorer than the controls for both the physical and mental component summary scales of the SF-36, reflecting limitations in physical ability and psychological distress. Such reductions in scores of SF-36 seem to be a common finding in people with chronic musculoskeletal diseases [61,62]. The control group scored about the same as the Norwegian normative values for both the physical and mental component summary scales of the SF-36. The WAD group displayed moderate physical disability due to neck pain as measured by the NDI. The mean score for the NDI are comparable to the participants with chronic WAD grade I-II in a series of studies [4,7,43,62,63], all displaying absolute NDI values very similar to our study (20–25.6). The findings in the present study should be treated with some caution due to the limited number of observations in this study. However, despite the moderate sample size, we found a number of statistically significant group differences. These findings were also seen across four different movements which further strengthens the findings.

### Conclusion and clinical implications

During simple, relatively unconstrained head movements, participants with chronic WAD move with less velocity and displacement compared with healthy controls. When taking these variables into consideration we found no difference in either rmsEMG amplitude or movement smoothness between the groups. People with chronic WAD do not seem to display signs of altered motor control patterns during unconstrained dynamic head movements. We suggest that while reductions in movement velocity and displacement are robust changes and may be of clinical importance in chronic WAD, movement smoothness of unconstrained dynamic movements is not.

## Abbreviations

A.u.: Arbitrary units; EBN: Extension back to the neutral head position; EFN: Extension from the neutral head position; FABQ: Fear avoidance beliefs questionnaire; FBN: Flexion back to the neutral head position; FFN: Forward flexion from the neutral head position; M: Maximum movement speed; NDI: Neck disability index; NJC: Normalized jerk cost; NP: Neutral head position; P: Preferred movement speed; rmsEMG: Root mean square electromyography; S: Slow movement speed; SCM: Sternocleidomastoid; SF-36: Short form-36; WAD: Whiplash associated disorders.

## Competing interests

The authors declare that they have no competing interests.

## Authors’ contributions

HV designed the study, processed the data, performed statistical analyses, drafted and revised the manuscript and participated in data sampling. ESB sampled the data, and participated in the design of the study and in the drafting of the manuscript. KL performed statistical analyses, participated in data processing and revised the manuscript. SRE sampled the data and participated in drafting the manuscript. NKV designed the study, participated in data processing and revised the manuscript. All authors read and approved the final manuscript.

## Pre-publication history

The pre-publication history for this paper can be accessed here:

http://www.biomedcentral.com/1471-2474/14/314/prepub

## Supplementary Material

Additional file 1 Data for movements at preferred speed with an additional load of 25% of head mass. Click here for file

Additional file 2 Methodological description. Click here for file
