# Supplementary material for: Muscle activity and head kinematics in unconstrained movements in subjects with chronic neck pain; cervical motor dysfunction or low exertion motor output?
Source: BMC Musculoskelet Disord. 2013 Nov 4;14:314. doi: 10.1186/1471-2474-14-314 (PMC3840692; doi:10.1186/1471-2474-14-314)
Supplement: Additional file 1 — Data for movements at preferred speed with an additional load of 25% of head mass. [file 1471-2474-14-314-S1.doc]

**Additional file 1**

**Data for movements at preferred speed with an additional load of 25% of head mass**

**Methods**

Following the movement tests under different speed conditions, subjects completed the movements in the four directions with additional loading corresponding to 25% of the head mass at their preferred speed only (P25). Modified pilot helmets (HGU-55/P, Gentex) were worn as a base for the supplementary head loading. Load plates, manufactured from polytetrafluoroethylene (PTFE), were put on PTFE -bars attached to the helmet on an axis parallel to the frontal plane passing approximately through the combined center of gravity of head and helmet. The center of gravity were calculated for the helmet and for 11 subjects according to the procedures of [McConville [1]](#_ENREF_1) and [Young [2]](#_ENREF_2), and projected on calibrated 2D profile images. The weighed combined center of gravity was calculated and marked on the 2D images of the helmet. The PTFE bars were then attached to each side of the helmet at this point. Measurements were completed using image software (ImageJ, version 1.37; Rasband, W.S, National Institute of Health). The chronic WAD group received an extra total load of a mean of 1.08 ± 0.1 kg (24.9 ± 0.9% of head mass). The corresponding value for the control group was 1.14 ± 0.08 kg (25.1 ± 1.2%). Kinematic and electromyograpic data were analyzed using the same procedures as described in the main document.

**Results and discussion**

Head kinematics with an additional head mass of 25% (1.1 ± 0.09 kg) were examined at the P speed only (supplementary table 1), and for both groups, the kinematics of these movements were remarkably similar to those of the unloaded condition. Thus, the statistical differences found between groups using two sample t-tests were also similar to those found for the unloaded P speed condition. After controlling for movement velocity and displacement using GLM analysis, there were no differences between the groups in NJC or number of submovements, with the exception of the FBN movement where the statistical difference for the number of submovements persisted (p < 0.01).

Differences between groups in muscle activity in the accelerative phase were found between the groups in the extra load condition in both the agonist and antagonistic muscles in the FBN direction (all p < 0.05, supplementary table 2). Additionally, increased antagonistic muscle activity in the control group as compared to the WAD group was found in the deceleratory phase in the FBN direction (p < 0.05). No differences were found between the groups in the EBN direction for the extra load condition. After using displacement and velocity as covariates, there were no significant differences in muscle activity between the groups for any muscle in any movement direction for the loaded test condition. In conclusion, the completion of movements with an additional 25% extra load was similar to that of the unloaded condition in both groups.

**References**

1. McConville, J.T., T.D. Chuchill, I. Kaleps, C.E. Clauser, and J. Cuzzi, *Anthropometric relationships of body and body segment moments of inertia*. 1980, Aerospace Medical Research Laboratory, Wright-Patterson Air Force Base: Dayton, Ohio.

2. Young, J.W., R.F. Chandler, C.C. Snow, K.M. Robinette, G.F. Zehner, and M.S. Lofberg, *Anthropometric and mass distribution characteristics of the adults female* 1983, FAA Civil Aeromedical Institute Oklaoma City, Oklahoma.

**Table S1.** Average (SD) kinematic and movement smoothness data for the extra load condition (P25) in the four movement directions for the chronic WAD (n=15) and control (n=15) group. Group differences are tested by two-sample t-tests. Statistically significant different from the control group; * p < 0.05, ** p < 0.01.

**EFN FBN FFN EBN**

WAD Control WAD Control WAD Control WAD Control

Displacement (°) 34.5 (15.2)** 55.6 (19.4) 39.1 (16.9)** 62.5 (20.9) 41.6 (14.1) 47.7 (8.3) 43.9 (14.3) 50.9 (11.1)

Duration (s) 2.29 (2.67) 1.56 (0.46) 1.79 (0.93) 1.35 (0.38) 1.65 (0.88) 1.31 (0.46) 1.73 (0.81)* 1.22 (0.25)

Peak vel. (°/s) 45.2 (32.5)* 70.4 (30.8) 51.8 (35.1)** 95.2 (36.1) 60.0 (38.3) 77.2 (30.3) 59.5 (31.0)* 84.1 (28.2)

Average vel. (°/s) 23.5 (16.3)* 38.3 (16.2) 27.3 (19.0)** 48.1 (17.0) 32.0 (20.0) 41.6 (16.9) 29.6 (14.3)** 43.7 (12.5)

Peak acc. (°/s/s) 172.0 (140.0) 257.0 (156.1) 215.0 (179.4)** 391.1 (199.6) 216.3 (158.1) 286.3 (174.7) 250.8 (181.6)* 366.5 (188.9)

Peak dec. (°/s/s) -147.1 (140.2)* -203.1 (122.0) -160.7 (120.8)** -251.0 (128.0) -235.5 (208.6) -304.9 (191.6) -166.1 (120.3) -223.7 (109.8)

NJC (a.u.) 808.0 (2784.0) 52.1 (28.6) 129.6 (179.0)* 41.7 (19.6) 96.5 (106.3) 49.0 (34.2) 101.2 (111.7)** 33.5 (11.4)

Submov. (no.) 6.5 (12.6) 2.3 (1.2) 4.2 (3.8)** 1.6 (0.9) 3.6 (3.2) 2.1 (1.2) 3.0 (2.7)* 1.4 (0.6)

Abbreviations; Vel. – velocity, Acc – acceleration, Dec. – deceleration, NJC – normalized jerk cost, a.u. – arbitrary units, Submov. – submovements, no. – number, NP – neutral head position, EFN – extension from NP, FBN – flexion back to NP, FFN – flexion from NP, EBN – extension back to NP.

**Table S2**. Average (SD) EMG amplitude values (%) for the SCM and splenius muscle for the WAD (n=15) and control (n=15) groups for the accelerative and decelerative phases during loaded movements in the FBN and EBN movements. Note the differences in the ordering of muscles between movement directions. Statistically significant differences between the groups were found by two-sample t-tests; * p < 0.05, ** p < 0.01. These statistically significant differences between the groups vanished when using displacement and velocity as covariates in the analysis of covariance.

**Accelerative phase Decelerative phase**

Direction Muscle Chronic WAD Control Chronic WAD Control

FBN SCM 86.1 (92.1)** 153.3 (69.5) 28.9 (27.4) 28.0 (23.0)

Splenius 31.5 (46.2)** 71.8 (66.2) 8.6 (8.0)* 20.6 (22.4)

EBN Splenius 100.9 (101.0) 132.2 (116.0) 23.9 (19.3) 21.6 (14.0)

SCM 11.3 (12.5) 6.5 (10.1) 6.1 (7.0) 4.9 (6.5)

Abbreviations; NP – neutral head position, FBN – flexion back to NP, EBN – extension back to NP, SCM – sternocleidomastoid.
