# Supplementary material for: Muscle activity and head kinematics in unconstrained movements in subjects with chronic neck pain; cervical motor dysfunction or low exertion motor output?
Source: BMC Musculoskelet Disord. 2013 Nov 4;14:314. doi: 10.1186/1471-2474-14-314 (PMC3840692; doi:10.1186/1471-2474-14-314)
Supplement: Additional file 2 — Methodological description. [file 1471-2474-14-314-S2.doc]

**Additional file 2 methodological description**

**Electromyography**

***Muscles and sensor placement.*** After localizing and inspecting the areas for placement of the EMG sensors, the parallel electrode bars of the sensors were oriented at a right angle to the fascicles of the muscles as visualized by ultrasound imaging. In accordance with [Joines [1]](#_ENREF_1), we found that some of the participants did not display a gap between the SCM and trapezius muscles large enough to access the deeper lying splenius muscle with the entire sensor. In those cases we chose to place the sensor closer to, or in a few cases one of the electrode bars above, the trapezius muscle. This was done to avoid possible cross-talk from the SCM, as prior pilot experiments had shown that the SCM muscles were more active during head movements than the trapezius. EMG activity was also recorded bilaterally from the upper trapezius muscle using previous suggested sensor placement . In accordance with previous studies we found very low EMG activity for this muscle (not exceeding 8.5% in the EBN movement in the M speed test) in both groups and have therefore not reported the data.

***Data sampling and EMG normalization procedures.*** Baseline EMG was recorded for each muscle while sitting with the head in the NP as defined in the methods section of the article. The median running rmsEMG (window length 100 ms, 99 ms overlap) obtained for the lowest 2 seconds was defined as the baseline value. The procedures for obtaining reference contractions of the different muscles were as follows; For the SCM muscle, the participants sat with back and head support adjusted to 45° to the vertical plane. Participants lifted and held the head slightly off the head support. For the splenius muscles, participants lay horizontally on their side on a bench with head support that was adjusted to align the cervical and thoracic column in all three planes. The participants then lifted and held the head slightly off the head support in the vertical direction. The participants did three isometric contractions for each test. Each contraction lasted about 10 s and the first and last 2 s epochs were removed from analysis. The median running rmsEMG (100 ms window/99 ms overlap) was calculated for each muscle and the median value of the three trials was used as reference signal.

**References**

1. Joines, S.M., C.M. Sommerich, G.A. Mirka, J.R. Wilson, and S.D. Moon: **Low-level exertions of the neck musculature: a study of research methods***.* *J Electromyogr Kinesiol,* 2006. **16**(5):485-497.

2. Jensen, C., O. Vasseljen, and R.H. Westgaard: **The influence of electrode position on bipolar surface electromyogram recordings of the upper trapezius muscle***.* *Eur J Appl Physiol,* 1993. **67**(3):266-273.

3. Schuldt, K. and K. Harms-Ringdahl: **E.m.g./moment relationships in neck muscles during isometric cervical spine extension***.* *Clin Biomech,* 1988. **3**(2):58-65.

4. Keshner, E.A., D. Campbell, R.T. Katz, and B.W. Peterson: **Neck muscle activation patterns in humans during isometric head stabilization***.* *Exp Brain Res,* 1989. **75**(2):335-44.

5. Benhamou, M.A., M. Revel, and C. Vallee: **Surface electrodes are not appropriate to record selective myoelectric activity of splenius capitis muscle in humans***.* *Exp Brain Res,* 1995. **105**(3):432-8.
